# Variability and the form–function framework in evolutionary biomechanics and human locomotion

**DOI:** 10.1017/ehs.2022.28

**Published:** 2022-07-07

**Authors:** Alison A. Murray

**Affiliations:** Department of Anthropology, University of Victoria, Cornett Building Room B228, 3800 Finnerty Road, Victoria, BC, Canada V8P 5C2

**Keywords:** Biomechanics, human evolution, bipedalism, kinematics, functional morphology

## Abstract

The form–function conceptual framework, which assumes a strong relationship between the structure of a particular trait and its function, has been crucial for understanding morphological variation and locomotion among extant and fossil species across many disciplines. In biological anthropology, it is the lens through which many important questions and hypotheses have been tackled with respect to relationships between morphology and locomotor kinematics, energetics and performance. However, it is becoming increasingly evident that the morphologies of fossil hominins, apes and humans can confer considerable locomotor diversity and flexibility, and can do so with a range of kinematics depending on soft tissue plasticity and environmental and cultural factors. This complexity is not built into traditional biomechanical or mathematical models of relationships between structure and kinematics or energetics, limiting our interpretation of what bone structure is telling us about behaviour in the past. The nine papers presented in this Special Collection together address some of the challenges that *variation* in the relationship between form and function pose in evolutionary biomechanics, to better characterise the complexity linking structure and function and to provide tools through which we may begin to incorporate some of this complexity into our functional interpretations.

## Graphical abstract

**Figure:**
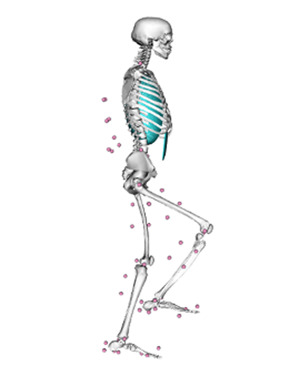

**Social media summary:** A skeletal structure can have many functions, giving flexibility but complicating functional interpretations from bone.

## 1. Central theme: integrating variability into the interpretation of functional morphology

The appearance of specialised bipedal morphology in the fossil record is one of the earliest indicators of the hominin divergence from the lineage leading to modern apes (Zollikofer et al., 2005). Understanding *how* and *when* human-like bipedal kinematics evolved is crucial to thinking about *why* hominin bipedalism evolved, and why only in hominins (Raichlen & Pontzer, 2021). To get at these fundamental questions in biological anthropology, we rely heavily on the characteristics of preserved skeletal elements to infer function and behaviour in life. This form–function conceptual framework, in which strong correlation between the structure of a particular trait and its function is assumed, is long-standing (Blits, 1999) and seminal not just in biological anthropology but in many scientific disciplines interested in anatomy and morphology. The form–function interpretive framework is the lens through which many important questions and hypotheses have been tackled in biological anthropology, ranging from why humans face such a tight fit during childbirth (Washburn, 1960) to the importance of endurance running in hominin evolution (Bramble & Lieberman, 2004). The form–function theoretical paradigm continues to be a crucial framework for understanding the origins of bipedalism, the selective factors shaping morphological variation and the impact of that variation on kinematics, energetics and locomotor performance. However, it has become increasingly evident that the morphologies of fossil hominins, apes and humans can confer considerable locomotor diversity and flexibility, and can do so with a range of kinematics depending on soft tissue deformation, plasticity and environmental and cultural factors. The nine papers in this Special Collection all highlight a range of innovative efforts to better consider this variability in the interpretation of functional morphology in the context of evolutionary biomechanics and human movement.

## 2. Mosaic morphology and the form–function paradigm

Typically, key skeletal differences between humans and other extant primates in areas like the pelvis, spine and foot (Williams & Russo, 2015; Holowka & Lieberman, 2018) have been interpreted as derived adaptations that increase efficiency in either terrestrial bipedalism or arboreal locomotion. However, increasing numbers of fossil hominins spanning millions of years of evolution and a wide geographic range all show evidence of mosaic morphology. Various combinations of arboreal and bipedal traits have been documented among the earliest hominins, like *Ardipithecus ramidus* (Lovejoy, Suwa, et al., 2009; Lovejoy, Latimer, et al., 2009), numerous australopithecine species (Stern & Susman, 1983; Williams et al., 2021; Dunmore et al., 2020; Georgiou et al., 2020; Meyer et al., 2017) and several members of the genus *Homo* (Pontzer et al., 2010; Brown et al., 2004; Détroit et al., 2019; Johanson et al., 1987; Lordkipanidze et al., 2007; Kivell et al., 2015). The widespread presence of mosaic morphology in the hominin fossil record suggests the presence of perhaps more than one strategy for meeting the biomechanical challenges of bipedal walking without sacrificing functional abilities in other locomotor modes as well.

Extant species help demonstrate this point with respect to variability in relationships between structure and functional parameters. Contemporary humans employ their bipedal walking anatomy in diverse ways, including running and vertical climbing, and to varying degrees depending on cultural variation, soft tissue plasticity, social learning and practice (Venkataraman, Kraft, Desilva, & Dominy, 2013; Venkataraman, Kraft, & Dominy, 2013; Wallace et al., 2022). For example, despite being well adapted for bipedal locomotion, the human ankle and foot can produce almost complete overlap with wild chimpanzees in range of motion during vertical tree-climbing (Venkataraman et al., 2013). Soft tissue plasticity developed over a lifetime of practice enables extreme ankle flexion well beyond what would cause injury or muscle tearing among non-climbing human populations (Venkataraman et al., 2013). Not only can humans be surprisingly good climbers, but all extant apes can and do occasionally move bipedally on the ground (Rosen, Jones, & DeSilva, 2022); facultative bipedalism is one of a range of locomotor modes used by apes, including suspensory locomotion, arboreal bipedalism and quadrupedalism, brachiation and knuckle-walking (Napier & Napier, 1967). All primates walking bipedally need to maintain balance and stability while on one leg in the stance phase, propel that leg forward during the swing phase and deal with impact forces when the foot strikes the ground again, all as efficiently and ‘cheaply’ as possible. However, their distinct anatomies pose different problems in doing so, requiring different solutions.

Much focus has been paid to key differences in the human pelvis relative to that of extant apes, for example our shorter and more laterally oriented iliac blades and shorter ischium (Marchal, 2000). These pelvic traits enable humans to prevent hip adduction during the single-leg stance phase of bipedal locomotion, and to maximise hip hyperextension before toe-off and leg swing (O'Neill et al., 2022). Based on the assumption that similar problems and solutions would have existed for hominins, shorter laterally oriented iliac blades and a shorter ischium are considered key derived adaptations indicative of habitual bipedalism in fossils (Stern & Susman, 1983; Lovejoy, Suwa, et al. 2009). However, fossil hominins exhibit a range of variation in ischial length, relative pelvic breadth and iliac blade orientation, amongst others (Kozma et al., 2018; Lovejoy, Suwa, et al., 2009), as well as mosaic sets of traits in key areas like the spine, lower limb and foot (Lovejoy, Latimer, et al., 2009; McNutt, Zipfel, & DeSilva 2018; Zipfel et al., 2011; Williams et al., 2021; Meyer et al. 2017). All of these skeletal structures are embedded in an intricate complex of soft tissues (muscle, tendon, ligament, fascia, aponeuroses, etc.), each with its own mechanical properties, contributions to overall function and associated costs. Variation in soft tissue can thus shape the function possible from a given skeletal structure and the overall cost of locomotion, yet this influence is not necessarily detectable from skeletal structure alone (e.g. Venkataraman, Kraft, and Dominy, 2013).

The deformation of soft tissues contributes a substantial amount of the work done during bipedal locomotion (Zelik & Kuo, 2010), passive work that then does not need to be actively performed by the muscles, saving energy. Dissipating energy at heel-strike, and powering toe-off, are two challenges that any species walking bipedally faces: among humans, meeting these challenges through soft tissue deformation seems to be the solution converged upon during our evolutionary history. Deformation of soft tissue structures like muscle fibers and tendons, ligaments of the ankle and foot, heel fat pad and plantar fascia probably perform most of the negative work done when the foot collides with the ground, reducing the extent to which the muscles must actively contribute (Zelik & Kuo, 2010). When stretched sufficiently, the elastic recoil of soft tissue structures can also contribute much of the positive work needed at toe-off in humans (Hof, Geelen, & Van den Berg, 1983; O'Neill et al., 2022; Sawicki, Lewis, & Ferris, 2009; Zelik, Huang, Adamczyk, & Kuo, 2014; Zelik & Kuo, 2010). The contribution of soft tissue deformation to the work of running is even greater than in walking: for the body as a whole, soft tissue deformation accounts for more than 25% of all negative work during the stance phase, and provides ~17% of the positive work for rebound when running (Riddick & Kuo, 2016). Maximising soft tissue deformation may thus have been an important part of the selective environment acting on key locomotor regions of the hominin skeleton. There is some suggestion that the shorter ischium of humans relative to chimpanzees (Aiello & Dean, 1990) enables greater energy storage in the anterior hip soft tissue, the release of which may contribute up to 35% of the work required at toe-off and leg swing in humans (O'Neill et al., 2022). These soft tissues do not preserve, and their contribution to locomotion among fossil hominins is difficult to estimate.

Another challenge of bipedal locomotion that any species must face is keeping the pelvis stable during the single-support phase. This must be achieved regardless of variation in pelvic traits like overall breadth, iliac blade size and the orientation of the ilia and ischia, variation resulting from the particular set of obstetric, locomotor and thermoregulatory challenges in a given species living in a given environment (Gruss & Schmitt, 2015). Differences in pelvic anatomy and musculature between humans and chimpanzees pose different problems and involve different solutions when it comes to the shared problem of maintaining pelvic stability on one leg. As such, they exhibit two distinct mechanical environments about the hip during the single-support phase of bipedal walking: chimpanzees utilise a mechanism involving large internal rotation and adduction moments at the hip to keep the pelvis level, whereas humans employ a strong hip abduction moment (O'Neill et al., 2022). Human-like walking mechanics thus cannot be applied to a chimpanzee, and we cannot necessarily assume that they are applicable to fossil bipeds either, given the wide range of variation in their pelvic traits (Lovejoy, Suwa, et al., 2009; Lovejoy, Heiple, & Burstein, 1973; Stern & Susman, 1983; Lovejoy, 2005; Berger et al., 2015; Jungers et al., 2009; Berge & Goularas, 2010; Berge, 1984). This variation not only spans the range of variation seen in extant species, but it occasionally represents unique combinations of features as well (e.g. Berger et al., 2015).

A huge challenge with interpreting skeletal variation in the past using the form–function paradigm becomes evident when considering this mosaic morphology and hominin variability: a given function might be possible with a range of structurally different bones, and structurally similar bones could function differently depending on the overlying soft tissue. In their contribution to this Special Collection, Juliet McClymont et al. (2022) argue that a shift is needed in bioarchaeology and paleoanthropology, from a paradigm based on form–function to one based instead on degeneracy. Degeneracy is a mechanism of adaptation within a system whereby structurally different bones can function in a similar way, while simultaneously each having a range of independent functions (McClymont, Davids, & Crompton, 2022). Viewed through the lens of degeneracy, mosaic morphology becomes less of a confusing contradiction: for example, the mosaic nature of early hominin foot morphology can be viewed as a flexible and highly degenerate system well suited for both arboreality and terrestrial bipedalism. The structurally different bones between species need not be viewed so much as specialised for dichotomous functions, but more so as part of a flexible system used to produce a shared function – bipedal walking – as well as other independent functions, like grasping or climbing. Importantly, this complexity in the relationship between structure and function is not necessarily discernible from skeletal remains alone.

### 2.1. Comparative behavioural approaches to thinking about the origins of bipedalism

The closest possible correlate of potential locomotor modes in the past is locomotion among extant species of apes and humans. As such, models of how and when hominin bipedalism emerged are frequently based on consideration of the characteristics and locomotor modes of extant apes. A long-standing and influential model sees the last common ancestor (LCA) of hominins and great apes as similar to an African ape, with a significant terrestrial knuckle-walking component to its locomotion (Richmond, Begun, & Strait, 2001; Washburn, 1967). When African apes move bipedally, they do so with a bent-hip–bent-knee posture that generates substantial torque about the flexed hip and knee that must be counteracted by sufficient muscle force to prevent the leg collapsing (Sockol, Raichlen, & Pontzer, 2007). Humans walk bipedally with an extended leg posture, reducing the muscle forces required and thus the associated energetic costs (Pontzer, Raichlen, & Sockol, 2009), providing a selection pressure that could theoretically drive the evolution of human-like bipedal walking from a knuckle-walking African-ape-like ancestor. The locomotion of Asian apes like hylobatids (gibbons and siamangs) and orangutans often involves arboreal bipedalism, or walking upright along the top of branches with an orthograde posture and an extended lower limb (Thorpe, Holder, & Crompton, 2007). Alternative models of the LCA that emphasise arboreal bipedalism, like those based on a hylobatian LCA (Tuttle, 1974), see an easier transition to hominin bipedality if the LCA is already frequently using a bipedal posture and extended leg in the trees. The kinematics and locomotion of the LCA is thus a matter of keen debate: was it predominantly terrestrial or arboreal? When it stood upright, did it adopt the flexed with a bent-hip–bent-knee posture of an ape, or the more extended limb posture of a human?

Seeking answers to these questions from skeletal remains alone is not straightforward; as mentioned above, structures specialised for a variety of locomotor modes can function in multiple and overlapping ways that are not necessarily reflected in bone structure alone. In this Special Collection, Rosen and colleagues (2022) approach the question of the LCA's locomotor mode through the examination of bipedal frequency in extant zoo-housed primate taxa. The most arboreal among extant primates, hylobatids and orangutans, both utilise an orthograde posture and frequent arboreal bipedalism involving an extended leg posture. However, hylobatids have an additional trait key to human bipedalism: a long and flexible lumbar spine. It has previously been argued that these traits, shared by two taxa who now have distinct primary locomotor modes (bipedalism and brachiation), reflect the ancestral condition (e.g. hylobatian model; Tuttle, 1974), but Rosen and colleagues explore whether or not it is arboreal individuals with these traits already who are most likely to move bipedally when on the ground. Among extant apes housed in zoos, they observed bipedal behaviour on the ground more often, and for longer per bout, among hylobatids than bonobos, chimpanzees, gorillas, orangutans or cercopithecines (Rosen et al., 2022). Of the 93 hylobatid individuals observed, bipedal bouts were documented among 83% of them, on average almost twice a day, and more than 35% of these bouts involved eight or more bipedal strides. Although strong selection could feasibly favour the evolution of obligate bipedalism from any of the observed taxa, as they all exhibited some bipedal behaviour, only the hylobatids already exhibited both an orthograde posture and a long and flexible lower back. An LCA with these traits as well may have been similarly predisposed to bipedal behaviour, in which case the orthograde posture and long lumbar spine of humans and hylobatids would be ancestral, and the short stiff lumbar spine and more pronograde posture of knuckle-walking apes would be the derived condition.

This behavioural work stimulates many interesting questions about the nature of the body form from which habitual bipedalism emerged, but also again highlights one of the main issues with the form–function paradigm that forms the central theme of this Special Collection: different skeletal structures can perform a given function reasonably well and can also simultaneously be used for a set of other independent functions. Even if a perfectly preserved fossil ape from exactly the right time was discovered, immediately before the divergence between hominin and great ape branches, and it had a long, flexible lumbar spine and seemingly an orthograde posture, this morphology can clearly be used in locomotor modes as diverse as habitual bipedalism and brachiation. The presence of one trait alone is not enough; approaches are necessary that can consider complexes of traits working together in flexible ways, as well as reconstruct soft tissue's role in both enabling functional diversity and generating structural variation in bone.

## 3. Bringing flesh upon you: reconstructing muscle's influence on bone structure and function

In bioarchaeology, inter- and intra-population variation in limb bone structural properties is often the starting point for informing inferences about mechanical loading, and thus activity, in the past (Ruff & Hayes, 1983; Ruff et al., 2015; Macintosh, Pinhasi, & Stock, 2014; Bridges, Blitz, & Solano, 2000; Sládek, Berner, Sosna, & Sailer, 2007; Stock & Pfeiffer, 2001). A significant amount of the internal force to which limb bones are subject comes from the contraction of skeletal muscles (Burr, 1997; Robling, 2009), forces that can rotate joints about an axis or centroid, thus creating moments (torques) (Stewart & Hall, 2006) that lever the bones of the skeleton around to both generate desired movement and prevent it when undesired. These muscle-related moments have been associated with patterns of bone functional adaptation in athletes relative to non-athletes (Nikander, Sievänen, Uusi-Rasi, Heinonen, & Kannus, 2006; Schipilow, Macdonald, Liphardt, Kan, & Boyd, 2013), explaining large proportions of variance in bone structural characteristics. For example, a proxy for muscle moment explained almost 50% of the variance in proximal tibial bending/torsional rigidity among a group of young adult female runners and soccer players, and over 80% of the variance in midshaft femoral bending/torsional rigidity in a group of young adult female control subjects (Murray & Stock, 2020). The 30% difference in the explanatory power of muscle moments for bone strength among athletes relative to non-athletes highlights the importance of external forces, in addition to internal ones, in contributing to moments about a joint. One such external force is the ground reaction force (GRF) exerted on the limb when the foot hits the ground during locomotion (Stewart & Hall, 2006). Athletes experience more and larger external moments than do non-athletes, and thus external moments probably explain more substantial proportions of variance in their bone strength indices. This is particularly true among athletes in sports where GRFs would be especially large or multi-directional, like soccer, triple jump and racquet sports. These athletes exhibit much greater enhancement of bone strength indices relative to controls than do athletes in sports involving high *muscle* magnitudes (internal forces) but negligible ground impact and GRFs (external forces; e.g. powerlifting, rowing, swimming, cycling; Macintosh & Stock, 2019; Niinimäki et al., 2017, 2019; Nikander et al., 2006).

When making behavioural inferences from bone structure in the past, we do not necessarily know the moments required to move a particular joint during various activities practised, and we do not know the relative contribution of external and internal forces in generating these moments. Further, bone structure reflects adaptation to the external and internal forces it experiences, regardless of whether those come from physical activity or not (for example, body mass loads weight-bearing bones). Population-specific equations have been developed to estimate body mass from skeletal remains (Ruff, 2000; Squyres & Ruff, 2015; Chevalier et al., 2018; Ruff et al., 2012; Ruff, Trinkaus, & Holliday, 1997) in order to remove its effect when interpreting activity from bone structure. However, the remaining functional signal in bone still reflects a range of external and internal forces, both of which can explain large portions of the variance in bone structure among humans, and neither of which are directly known in the past. As such, behavioural inferences are typically broad (more vs. less mobility, more vs. less asymmetrical etc.) and linked to archaeological or historical evidence of more specific activities when possible. Reconstructions and interpretations of the anatomy and behaviour of past hominins and humans would greatly benefit from an improved ability to estimate the external and internal forces, and the coordinated patterns of muscle activity, which may have been associated with particular activities in the past.

**Figure 1.**
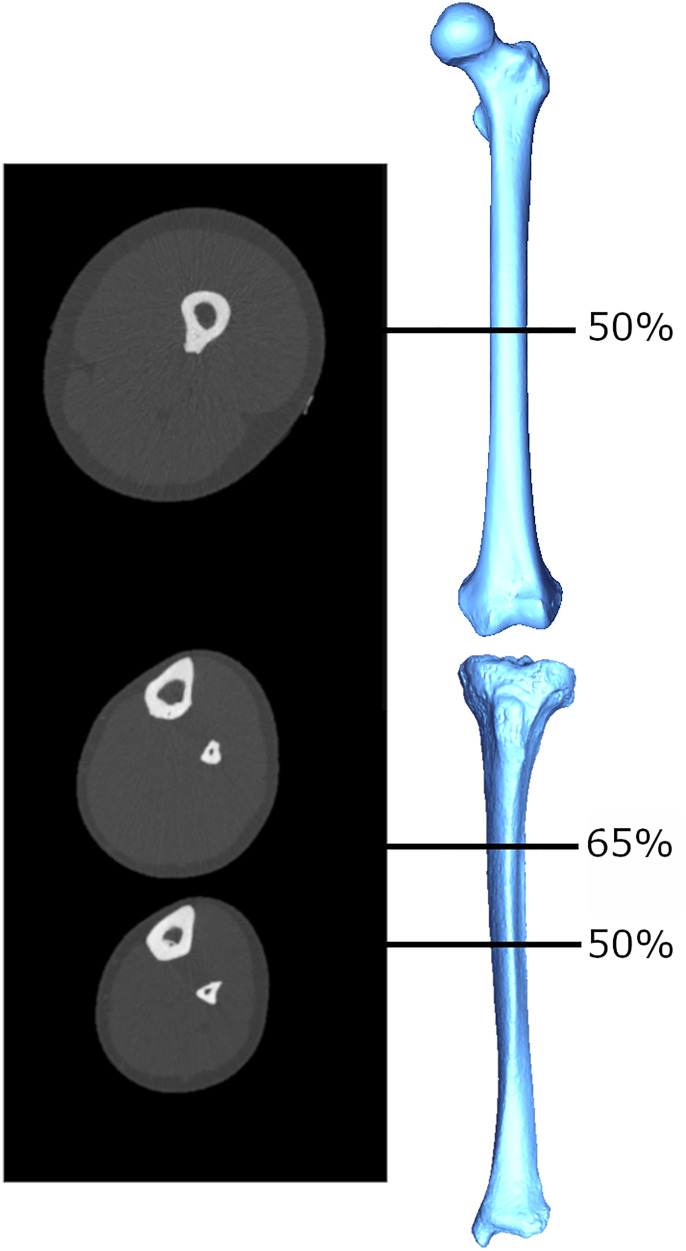
Example cross-sectional images obtained using peripheral quantitative computed tomography (pQCT), from which limb bone CSG properties and MCSAs are quantified. The mid-thigh image is taken at 50% of femoral length, and the lower leg images are taken at 65% and 50% of tibial length. Greyscale coloration represents tissue density: white (most dense) = bone, light grey = lean mass, dark grey (least dense) = fat mass.

There have been several attempts in biological anthropology to estimate muscle size from bone structural properties. These attempts have their basis in the roughly proportional relationship between a muscle's cross-sectional area (MCSA) and its force production that has been documented among living humans (Maughan, Watson, & Weir, 1983). The MCSA exhibits strong correlations with measures like grip strength (Frank, Lorbergs, Chilibeck, Farthing, & Kontulainen, 2010) and explains significant amounts of variance in limb bone cross-sectional bone areas and indices of bone strength (Frank et al., 2010; Heinonen, Mckay, Whittall, Forster, & Khan, 2001; Lorbergs, Farthing, Baxter-Jones, & Kontulainen, 2011; Macdonald, Kontulainen, Petit, Janssen, & McKay, 2006; Rantalainen, Nikander, Kukuljan, & Daly, 2013). Relationships between MCSA and commonly used cross-sectional geometric (CSG) properties in the upper limb bones (Shaw 2010; Slizewski, Onau, Shaw, & Harvati, 2013) have been explored and used to estimate forearm MCSAs for a sample of Neolithic humans (Slizewski, Burger-Heinrich, Francken, Wahl, & Harvati, 2014). However, the accuracy with which MCSAs predicted CSG properties were highly variable: percentage prediction errors ranged from 54% to as high as 85%, and percentage standard errors from ~8 to 19%, depending on sport, age, sex and slice location. This is potentially reflecting, at least in part, the need for an approach that considers functional relationships between bone and muscle complexes active during particular movements. The estimation of MCSA from CSG properties in the studies above was attempted within a given computed tomography (CT) slice location, either at the midshaft (Shaw, 2010; Slizewski et al., 2013) or 65% of the bone length (Slizewski et al., 2013). However, the musculature immediately adjacent to the bone at these locations is not necessarily all of, or even the main, musculature that is generating moments and load on the bone at that slice location. For example, the slice taken at 65% of the forearm length does not capture the muscle bellies of the major elbow flexors and extensors (triceps brachii, biceps brachii, and brachialis; An, Hui, Morrey, Linscheid, & Chao, 1981). Examples of CT slice images for the lower limb are provided in Figure 1; muscle area at a given slice location is computed as the area of the light grey (muscle) tissue in cross-section, and individual muscles are not differentiated. Mid-lower leg CSG properties did not exhibit any significant relationship with MCSA from the same section location (Shaw, 2010), but alternatively have exhibited significant relationships with MCSA more proximally in the limb, particularly at the mid-thigh (Rantalainen et al., 2013; Murray & Stock, 2020). This is probably because many large muscles of the thigh insert on the lower leg bones, generating large bending moments about the knee joint that may result in tibial and/or fibular bone functional adaptation not explained well by calf muscle areas. In order to use limb bone structural geometry to predict or infer muscle size in the past and, following from that, force production, a functional approach is clearly necessary.

### 3.1. Mapping functional patterns of muscle activity

The strains experienced by a bone in the performance of a given behaviour are generated by a complex pattern of coordinated muscle activity within limbs and across joints. However, the direct *in vivo* measurement of bone strain produced during the performance of a particular task in humans is understandably uncommon (see review in Al Nazer, Lanovaz, Kawalilak, Johnston, & Kontulainen, 2012). Electromyography (EMG) has proven useful over the past decade in biological anthropology in assessing experimentally measured patterns of muscle activity during the performance of tasks important among past populations, to which patterns of bone functional adaptation thought to be generated by that task can be compared. Most work to this end thus far has focused on inferred skeletal signatures of key hunting and food-processing technologies, like querns, spears and projectile weaponry. Such research has provided support for behavioural interpretations linking changes in humeral asymmetry among European agricultural women to technological shifts in grain-grinding technology (Sládek, Hora, Farkasová, & Rocek, 2016), but it has also challenged influential and longstanding hypotheses about the role of underhanded spear thrusting (Churchill et al., 1996) in the pronounced humeral asymmetry among Neanderthals (Shaw, Hofmann, Petraglia, Stock, & Gottschall, 2012).

The development of projectile weaponry like the bow and arrow represented both a shift in cognitive complexity, being made and used only by *Homo sapiens*, and a key piece of the toolkit enabling human dispersal and adaptive success (Williams, Burke, & Lombard 2014; Shea, 2006). Being predominantly made from organic materials, archaeological evidence of archery usually relies on finding stone, bone or metal arrowheads. However, the use of the bow and arrow requires substantial muscle force production at the elbow and shoulder in the draw (dominant) arm, force known as the draw weight that, for composite and longbows in the past, is in the range 90–130 lb or 40–60 kg (Hardy, 1992; Karpowicz, 2007; Soar, 2006). The bow (non-dominant) arm must brace against these large forces to keep the arm straight, so archery may be detectable in asymmetrical humeral functional adaptation. Among the humeri of males thought to have performed archery, a pattern of relatively symmetrical responses to loading intensity (limb bone bending strength, external dimensions, total areas etc.) but asymmetrical responses to loading directionality (limb bone shape ratio) has been documented (Rhodes & Knüsel, 2005; Stirland, 1993). Whether or not the performance of archery actually *does* involve symmetrical upper limb load intensity but asymmetrical load directionality is unclear, however. In this Special Collection, Tabitha Dorshorst and colleagues (2022) utilised EMG analysis to investigate the patterns of muscle activity at the shoulder and elbow in both upper limbs of participants throughout the drawing of a bow. Peak percentages of maximum voluntary contraction in the muscles of the bow and draw arms were similar, but were generated by the joint agonist in one arm and its antagonist in the other arm (Dorshorst, Weir, Hamill, & Holt, 2022). This pattern of muscle activity about the shoulder and elbow during archery is consistent with signatures of symmetrical loading intensity overall but asymmetrical directionality inferred from skeletal remains.

### 3.2. Modelling activity-related external and internal forces

**Figure 2.**
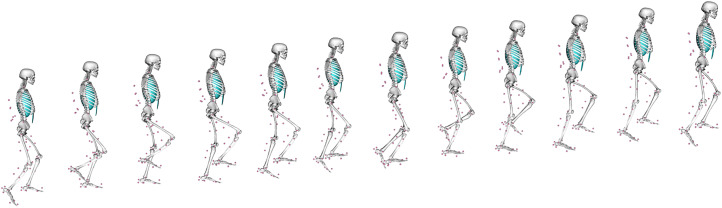
An example of a musculoskeletal model at different points in a stair-climbing simulation. Pink circles indicate the position of reflective motion capture markers placed at anatomical landmarks on the subject in life as they performed the movement.

Although useful, EMG does not directly measure internal bone loading, as this is very difficult to measure *in vivo*. A variety of other parameters are also difficult to measure *in vivo*, like muscle–tendon lengths, moment arms and soft tissue deformation, all of which affect forces, kinematics and costs during locomotion. Musculoskeletal (MSk) modelling provides a computational means of estimating such parameters during the performance of a given movement (Arnold et al., 2000; Wehner, Claes, & Simon, 2009; Baggaley et al., 2022), so is a powerful tool in trying to better understand locomotor kinematics in the past. Using a combination of motion equations and muscle force prediction equations, MSk techniques can determine a range of kinematic and soft tissue parameters that would exist within a subject when conducting any specific activity that can be virtually defined or measured experimentally (e.g. walking). To do so, an MSk model typically requires detailed information on a subject's musculoskeletal anatomy, from cadaveric dissection, CT imaging, or magnetic resonance imaging. These anatomical data are then paired with experimental data on gait and muscle parameters to create a representative model of a subject moving; an MSk model climbing stairs is presented in Figure 2. The greatest accuracy is achieved by collecting all input data from the same individual (Charles, Grant, D'Août, & Bates, 2020), although this is not always possible. Even with a full subject-specific dataset, MSk models are sensitive to error in bony landmark identification, muscle tension and muscle geometries, all of which affect the calculated muscle and joint moments and forces (Broyde et al., 2021; Martelli, Valente, Viceconti, & Taddei, 2015; Valente et al., 2014). Even just the removal of soft tissue during dissection can change muscle/tendon paths enough to introduce large errors in muscle via points in an MSk model (Synek et al., 2019).

Obviously, the sensitivity of even subject-specific models to estimation errors is a major challenge facing the effective use of MSk modelling among extinct fossil species, in whom bony preservation may be poor and muscle and gait variables are simply unknown. Existing efforts to simulate locomotor biomechanics in fossil hominins have faced difficulties in determining segment lengths, relative body segment inertial parameters, passive joint properties, muscle characteristics, muscle attachment locations and even the anatomy of entire limb segments that were missing (Nagano, Umberger, Marzke, & Gerritsen, 2005; Wang et al., 2004). In order to overcome these issues, assumptions must be made that extant human or non-human primate data would have been similar enough to act as an appropriate substitute. Human or ape muscle, joint and relative body segment parameters can then be scaled to a given fossil's dimensions, and their experimentally collected gait data used to simulate a fossil walking (Nagano et al., 2005; Wang et al., 2004). This practice can contribute to the already considerable disagreement that exists about hominin locomotion; for example, that australopithecines walked both with a flexed, bent-hip–bent-knee bipedal gait (Stern, 2000; Stern & Susman, 1983) and with an extended gait (Crompton, Yu, Weijie, Günther, & Savage, 1998; DeSilva et al., 2013; Raichlen, Gordon, Harcourt-Smith, Foster, & Haas, 2010), and that they walked bipedally both less efficiently than a human (Nagano et al., 2005; Pontzer et al., 2009; Wang et al., 2004) and similarly or even more efficiently (Sellers, Cain, Wang, & Crompton, 2005; Kramer, 1999; Kramer & Eck, 2000).

Considering reference data for something like muscle architecture from one species as representative of that of another is obviously not ideal, but is currently the only option available to us when considering extinct species. However, this is a problem even when modelling extant human kinematics: when collecting subject-specific datasets is not possible, most of the reference data for ‘generic’ human muscle parameters come from cadaveric dissections of elderly individuals (Klein Horsman, Koopman, van der Helm, Prosé, & Veeger 2007; Arnold, Ward, Lieber, & Delp, 2010; Rajagopal et al., 2016). These muscle parameters are probably not representative of most humans, as not only does muscle architecture (volume, length, cross-sectional area, fiber length, pennation angle, etc.) vary substantially, even among individual humans (Charles, Suntaxi, & Anderst, 2019), it changes with age (Moore et al., 2014; Narici, Maffulli, & Maganaris, 2008). Compared with using elderly generic muscle architecture data, averaged data collected from young, healthy individuals (Charles et al., 2019) does predict experimentally measured subject-specific isokinetic muscle torques to a higher degree of accuracy (Charles et al., 2020). However, even generic data from *young* adults still introduced error relative to subject-specific muscle data for some movements at the ankle, knee and hip. In this Special Collection, Patricia Kramer, Feuerriegel, Lautzenheiser, and Sylvester (2022) test the extent to which variation in the muscle architecture parameters of functional groupings of lower limb muscles influenced joint reaction force and muscle force estimates in complex movements. Kramer and colleagues modified a previously existing baseline MSk model to the size and proportions of 10 healthy volunteers, to which subject-specific motion and force data, and cadaveric muscle architecture parameters, were applied. The resulting joint and muscle forces of these models were compared with the same models but with generic young adult muscle architecture parameters applied instead (Charles et al. 2019). Both across models and among individuals, muscle force patterns from the large lower limb functional muscle groups remained consistent across the gait, despite variation in the muscle architectural data used. However, the magnitude of those forces was affected by muscle parameter choices, with muscle and joint reaction forces varying as much as 15% depending on which muscle architecture dataset was applied. For some purposes, such as exploring shape differences in joint morphology among groups, this variation that muscle parameter choices induced in the resulting joint reaction forces may pose problems. In these instances, it is particularly important that the external kinematics, kinetics and muscle parameters used when comparing models are kept consistent (Kramer et al., 2022).

## 4. Revising traditional models of form, function, and energetics

Although MSk models are becoming more common in biological anthropology, traditionally the impact of skeletal traits on the kinematics and costs of walking and running have been estimated or measured using either mathematical models or experimental studies. The resulting estimates/data have formed the basis of many influential hypotheses in biological anthropology, despite their conclusions being highly dependent on the accuracy and representativeness of the model and experimental conditions. When you change these, you can completely upend the conclusions. For example, although the overall costs of running among humans are high compared with walking (Biewener, Farley, Roberts, & Temaner, 2004), it is typically thought that these costs are relatively independent of speed (Bramble & Lieberman, 2004; Carrier, 1984; Kram & Taylor, 1990; Margaria, Cerretelli, & Sassi, 1963; Menier & Pugh, 1968). This is an important contrast to the curvilinear relationship between cost and speed among quadrupeds, a relationship first reported by Hoyt and Taylor (1981) among ponies. This difference between the cost of transport curve in quadrupedal vs. human running forms the core of hypotheses proposing that endurance running was an important selection pressure shaping the evolution of the genus *Homo* (Bramble & Lieberman, 2004; Carrier, 1984). A linear cost of transport curve would allow a human hunter to sustain running speeds outside of the optimal speed of any quadrupedal prey (Carrier, 1984), and allow a persistence hunting strategy to be successful. There is no doubt that endurance running hypotheses have been extraordinarily influential in our view of human evolution, and have filtered into popular culture as well. Yet the models on which these costs of human and quadrupedal running are based suffer from the same issue as MSk models: they are built from a narrow set of input parameters that are not usually representative of variation in species- or group-level anatomy or of variation in the environmental, social and cultural conditions in which locomotion occurs in real life.

The curvilinear relationship between speed and cost of transport for quadrupeds first reported by Hoyt and Taylor (1981) for ponies was based on data from only three individual animals, and the subsequent extrapolation of these results as typical of all quadrupedal species occurred despite being refuted on multiple occasions (Taylor, Heglund, & Maloiy 1982; Taylor, Schmidt-Nielsen, & Raab 1970). Similarly, evidence of a linear relationship between human running speed and cost was derived sometimes from as few as two individuals, all male (Margaria et al., 1963). If these relationships are not accurate or widely applicable, our interpretation of the importance of endurance running in hominin activities becomes fuzzier. Interestingly, a carefully controlled experimental design aiming to test the fit of linear and curvilinear models to the energetic costs of human running at various speeds from 1.5 to 5.5 m/s among both males and females found a significantly better fit using a *curvilinear* model than a linear model, achieving an *R*^2^ of 0.998 (Steudel-Numbers & Wall-Scheffler, 2009). This stands in direct contrast to traditional models of independent running speeds and costs among humans on which endurance running hypotheses are based. Steudel-Numbers and Wall-Scheffler (2009) concluded that modern humans in fact do have an optimal running speed, and speeds above and below that are suboptimal in terms of energetic efficiency. If past hominins also had an optimal running speed, this would have increased the cost of persistence hunting so much that it may not actually have been feasible in the energy budget of past hominins.

Another of the most influential models that is fundamentally based on traditional models of locomotor efficiency and morphology is the Obstetrical Dilemma Hypothesis (ODH) (Washburn, 1960). The ODH posits that the female pelvis reflects an evolutionary trade-off where the locomotor efficiency of a narrow bipedal pelvis is compromised in order to accommodate the passage of a large-brained infant. The relationships between energetic efficiency and pelvic breadth on which this trade-off is based come from standard mathematical and biomechanical models of lower limb moments and forces during walking (Alexander & Jayes, 1978; Alexander, 1980; Cavagna & Kaneko, 1977; Lovejoy, Heiple, & Burstein, 1973; Ruff, 1995). These static models assume that external forces (GRFs) pass essentially vertically through the body's centre of gravity during the stance phase of walking, when the body is balanced on one leg – in other words, through the midline of the pelvis. As such, the moment arm of the GRFs that the hip abductors must counteract to keep the pelvis stable on one leg is taken as half of the bi-acetabular breadth of the pelvis (Lovejoy, Heiple, & Burstein, 1973; Ruff, 1995). Increasing bi-acetabular breadth (a wider pelvis) thus increases the GRF moment arm, and requires the hip abductors to exert more force to counteract it, increasing the cost of locomotion. However, more recent use of inverse dynamics paired with imaging, force plate and kinematic data from living subjects (Warrener, Lewton, Pontzer, & Lieberman, 2015) found that pelvic breadth was not associated with the cost of locomotion or hip abductor mechanics in either sex. Experimentally measured muscle activity among males and females walking on a treadmill actually seemed to suggest that a broader pelvis may *reduce* the cost of transport, by requiring less hip adductor and hamstring activity, regardless of speed or incline (Wall-Scheffler, Chumanov, Steudel-Numbers, & Heiderscheit, 2010). What this means for the ODH is a matter of debate, but the central tenet that a broader female pelvis reflects an evolutionary trade-off between locomotor efficiency and obstetric dimensions does not seem sufficient to explain variability in pelvic morphology, human gestation length or secular trends in cephalopelvic disproportion (Betti & Manica, 2018; Dunsworth, Warrener, Deacon, Ellison, & Pontzer, 2012; Fischer & Mitteroecker, 2015; Frémondière, Thollon, & Marchal, 2022; Gruss & Schmitt, 2015; Kurki, 2013; Ricklan, Decrausaz, Wells, & Stock, 2021; Wells, 2015; Wells, DeSilva, & Stock, 2012)

### 4.1. The impact of walking with others and carrying load

Not only does a broad pelvis not necessarily sacrifice locomotor efficiency, it in fact provides a distinct advantage in two particular conditions: when walking with others and when carrying load. Neither of these conditions were considered in traditional biomechanically modelled or experimentally measured costs of locomotion, yet in reality, humans often walk with other humans, some or all of whom may be carrying loads while doing so. Both walking with others and carrying loads can affect the cost of walking: carrying reproductive-sized loads (pregnancy, child) slows your optimal walking speed, makes it more costly and increases the energetic penalty for walking at a suboptimal pace (Wall-Scheffler & Myers, 2013), something which you may need to do when walking with other individuals (Wagnild & Wall-Scheffler, 2013). These challenges are cross-cultural, and several solutions seem to have been universally converged upon. All societies have some type of infant-carrying device to reduce the cost of load carrying, like slings, wraps, cradleboards, etc. (Dettwyler, 1988; Konner, 1976; Lewis-Williams & Bannister, 1991; van Hout, 2015), which can reduce the cost of carrying a child by 16% compared with carrying in the arms (Wall-Scheffler, Geiger, and Steudel-Numbers, 2007). Without an assistive device, the cost of carrying loads in the arms can be reduced instead by a broader pelvis, with savings of up to 12% (Wall-Scheffler et al., 2007). A broad pelvis also seems to confer greater speed flexibility (Wall-Scheffler & Myers, 2013), which is important in keeping locomotor costs down when walking in a group. When the conditions of load-carrying and group mobility are incorporated into models of the cost of bipedal locomotion, a new perspective on the notably broad pelves of both male and female fossil hominins emerges (Wall-Scheffler, 2012a; Wall-Scheffler et al., 2007; Wall-Scheffler & Myers, 2013).

In this Special Collection, Cara Wall-Scheffler (2022) explores the extent to which the elevated metabolic costs of carrying a child in a sling are affected by interactions between the terrain over which you are carrying and the position of the child on the body while doing so. Over a 1000 m section of natural undulating terrain, a dorsal-carrying position was the least expensive for both sexes relative to front- or side-loading, and was the only position that did not require the parent to slow down their overall walking speed (Wall-Scheffler, 2022). When encountering inclined sections of trail, parents carrying their children altered their kinematics regardless of load position, spending a significantly greater proportion of each stride in the stance rather than swing phase. This change was most dramatic for dorsal carrying, but for all positions, spending more time in stance phase probably enables better stability on a variable surface when carrying a precious cargo. In all positions (dorsal, side, front), mothers were able to carry their child more economically and more efficiently than fathers were, reflecting the relationship between a relatively wider pelvic breadth and reduced metabolic costs when carrying. The strategy that individuals employ to maximise their locomotor economy and efficiency is clearly highly variable, depending on their particular morphology, what they are carrying, where they are carrying it on their body, the terrain over which they are doing so and the kinematics that they employ. This complexity and also flexibility is simply not well captured in traditional biomechanical models of unloaded walking in fixed laboratory conditions.

### 4.2. The impact of substrate and terrain

Typically, locomotor costs when walking also tend to be derived from a subject moving not just alone and unloaded, but across a *hard flat surface*. On this substrate, long legs are associated with a lower cost of transport (Steudel-Numbers & Tilkens, 2004; Steudel-Numbers, Weaver, & Wall-Scheffler, 2007). This aspect of the relationship between morphology and energetics has been influential in interpreting some of the earliest and most distinctive phenotypic changes associated with our genus *Homo*: the elongation of the lower limb and increases in stature. Based on the costs of moving on flat, hard surfaces, very short stature and short legs would reduce stride length, and thus increase the cost of moving the body a given distance. However, this makes it very difficult to explain the convergent evolution of particularly small body size repeatedly in modern humans independently in multiple different populations around the globe (Perry & Dominy, 2009), as well as among other hominins too, for example SE Asian hominins like *Homo floresiensis* (Brown et al., 2004) and the recently discovered *Homo luzonensis* (Détroit et al., 2019). The answer may lie in the fact that not all substrates on which hominins and humans move(d) are hard and flat; when that condition changes, so too do the kinematics and energetic costs of locomotion.

Extinct and extant small-bodied populations have something in common: they live(d) in tropical rainforest, where vegetation is dense and the substrate is irregular and often compliant. Venkataraman et al. (2018) used experimental data from the Batek and the Tsimane, two small-bodied human populations who forage in rainforest, to demonstrate that step length is constrained in such dense vegetation, forcing taller individuals to walk slower than their optimum speed. The energetic costs associated with constrained step length in this environment were not directly measured, but in a laboratory setting, walking on a soft, rather than hard, substrate eliminates the significant relationship between leg length and a lower cost of transport (Charles, Grant, D'Août, & Bates, 2021). Locomotion on variable surfaces is also associated with a range of kinematic alterations that affect the work done by the muscles. On soft surfaces like sand, although less muscular effort is required to provide cushioning (Tung, Franz, & Kram, 2014), greater muscular work is required to control unwanted movement of the foot during footstrike and toe-off (Lejeune, Willems, & Heglund, 1998). The elastic recoil mechanisms of muscle–tendon units, crucial to human locomotor efficiency, are also dampened in substrates like sand (Lejeune et al., 1998), further increasing locomotor costs. Stride lengths tend to become shorter or more variable on soft or uneven surfaces (Dhawale & Venkadesan, 2021; Pinnington, Lloyd, Besier, & Dawson, 2005; Voloshina & Ferris, 2015; Voloshina, Kuo, Daley, & Ferris, 2013), and leg posture can become more flexed (Pinnington et al., 2005). These kinematic changes in relation to substrate typically increase the metabolic costs of locomotion (Davies & Mackinnon, 2006; Gast, Kram, & Riemer, 2019; Lejeune et al., 1998; Lussiana, Fabre, Hébert-Losier, & Mourot, 2013; Voloshina & Ferris, 2015; Voloshina et al., 2013), although not always (Dhawale & Venkadesan, 2021). However, the human locomotor system exhibits adaptability in key parameters like leg stiffness to accommodate changes in substrate and keep locomotor costs more stable: on uneven surfaces, runners reduce their joint stiffness (Dhawale & Venkadesan, 2021), but on soft substrates they increase it (Ferris, Louie, & Farley, 1998).

None of this variation in substrate, kinematics and costs is typically accounted for in traditional biomechanical models, highlighting the importance of research in more natural settings and conditions more typical of most of human evolution. In this Special Collection, Nick Holowka and colleagues (2022) explored how kinematics vary in relation to substrate in a natural forested setting, by assessing walking kinematics among the Tsimane of the Bolivian Amazon. Importantly, they quantified lower limb kinematics when walking not just across unbroken forest understory and an open hard-packed dirt field, but on forest trail as well. Trails are made and used by many human populations in heavily forested areas, probably because they provide some benefit to the energetic costs of locomotion and/or travel time, but these advantages are not well understood. Substantial changes in lower limb kinematics were evident when walking in unbroken forest compared with a hard-packed flat and open fields: the hips, knees and ankles were more flexed, the trunk more inclined, the foot swing higher and the foot strike flatter. These are in sharp contrast to the kinematics, like an extended leg, thought to be so crucial to energetically efficient bipedalism. Although walking with more flexed lower limb joints, inclined torso, a high-stepping gait and flatter foot strike would probably increase the cost of locomotion, in unbroken forest these changes help reduce the chances of tripping, lower the centre of gravity to aid balance and provide greater tactile sensitivity to the substrate, which are clear benefits when walking unshod on unstable and uneven surfaces. Forest trails mitigated some of the need for suboptimal walking kinematics, yet their construction and maintenance add their own energy costs, so more research is needed to determine how foraging strategies balance these costs and benefits in natural environments. However, for hominins moving on variable natural surfaces, some of them irregular, uneven, steep and/or slippery, it may be that the energetic advantages of bipedalism are more conservative, or at least more variable, than typical estimates of locomotor costs based only on walking over a flat open and hard substrate (Holowka, Kraft, Wallace, Gurven, & Venkataraman, 2022).

The importance of considering the environment in which a species moved when estimating the cost of locomotion is particularly crucial when interpreting the selective factors shaping diversity among hominins with wide geographic ranges, or between hominin species of African and Eurasian origins. For example, *Homo neanderthalensis*, a Eurasian species, had larger, broader bodies and relatively short legs and distal limb segments relative to *Homo sapiens*, a species of African origins (Holliday, 1997). As long distal limb segments, and relatively long limbs, are an advantage over flat terrain (Steudel-Numbers & Tilkens, 2004; Steudel-Numbers et al., 2007), these differences are estimated to have increased locomotor costs for Neanderthals by up to 30% (Steudel-Numbers & Tilkens, 2004). In contrast, short limbs, short distal limb segments and a large broad body provide a large volume of heat-generating tissue and minimal surface area through which to lose it, conferring a low surface-area-to-volume ratio that is a major advantage in cool conditions. Thus, the relative impact of thermoregulatory and locomotor selection pressures on the characteristic morphological differences between Neanderthals and humans is a matter of keen interest (Ocobock, Lacy, & Niclou, 2021; Tilkens, Wall-Scheffler, Weaver, & Steudel-Numbers, 2007; Weaver & Steudel-Numbers, 2005). The interpretation of Neanderthal morphology must also take into account that some of the Neanderthals’ Eurasian range was not open and flat, but mountainous. Biomechanical modelling of lower limb forces when walking over uneven and steep terrain suggests that, in these conditions, having a shorter distal limb segment can be advantageous (Higgins & Ruff, 2011). It is thus likely that Neanderthal limb and body proportions reflect optimisation to interrelating climatic and locomotion-related selection pressures. Among humans, this inter-relationship is probably even more complex, as we are a species of African origin now inhabiting every environment on the globe, and one for whom foraging strategies are extremely diverse and probably included a unique strategy: sustained running in the heat.

### 4.3. The interrelationship between locomotion and thermoregulation

Adaptations among humans enabling heat dissipation during sustained running are thought to have lifted one of the main constraints typically limiting the running abilities of other species (Carrier, 1984). Humans differ from most quadrupeds in the mechanisms through which we dissipate heat when running, including a prodigious ability to sweat that, unlike panting, has allowed evaporative heat loss over a large surface area independent of breathing (Taylor & Rowntree, 1973). The rate at which heat is lost is also maximised by our reduced body hair, enhancing both evaporative and convective cooling (Carrier, 1984). Ecogeographic patterning in surface-area-to-volume ratios of the body is also evident among humans globally, conforming broadly to Bergmann's and Allen's rules (Allen, 1877; Bergmann, 1847) and enhancing passive thermoregulation. Human physiology as well as body size, shape and proportions matter not just for locomotor efficiency while walking or running, but for our ability to efficiently dissipate the metabolic heat generated during locomotion in a particular environment as well. How are these costs reflected in the morphological variation of a single species with such a broad geographic range and diversity of foraging behaviours?

One unusual interdisciplinary way of investigating these questions is highlighted by a pair of papers originally published in 2019 and 2021 of which I was a part, which are included in this Special Collection as well. Together with Daniel Longman, Jay Stock and colleagues, we explored relationships between running performance and thermally optimised variation in body size, shape and proportions among ultrarunners racing in two extreme conditions: heat (southern Spain, Peruvian Amazon) and cold (Finnish Lapland, Nepalese Himalayas). Female athletes who successfully finished the hot races had significantly lower body mass and weight-for-height than those who finished the cold races, but significantly longer legs for a given height. On average then, female finishers in hot conditions had less tissue generating heat and more surface area through which to dissipate it, and the converse was true of finishers in cold conditions. Male athletes demonstrated similar patterns in relative leg length. However, participants racing in a given climate tended to have morphology more optimised for that climate in the first place, so these relationships could simply be reflecting self-selection. We thus also compared morphological traits among finishers and non-finishers in two consecutive years of the Al Andalus Ultimate Trail race, a multi-day 230 km semi-supported race. This race took place in hot, dry and relatively flat southern Andalucia at the height of summer, where daytime temperatures were in excess of 40°C. In these conditions, relationships between successful performance and morphology optimised for minimal heat generation and maximal heat loss were evident: all finishers had significantly longer legs for a given height than athletes who dropped out of the race (Longman et al., 2019), while female finishers were also significantly lighter and leaner than females who did not complete the race (Longman et al., 2021). Thus, the morphological trends demonstrated between finishers of hot and cold races are probably reflecting at least some influence of thermally adapted morphology on performance.

Physical exertion for 230 km in temperatures exceeding 40°C clearly places individuals at the extremes of their thermoregulatory tolerance, where any trait that passively dissipates heat with minimal cost is likely to be an advantage. Morphology may reflect the costs of locomotion, but not independently of the environment in which that locomotion is occurring. The body size and proportions that were associated with better performance in the hot, dry and flat race described above (only 7.1 km of ascent over 230+ km of running) cannot be assumed to be the case in other environmental contexts. One of the races from which we recruited participants in the ‘cold’ group, the Everest Trail Race, starts at 2370 m of elevation, and over the course of 153 km racers climb 13.5 km and descend 13 km (Longman et al., 2021). Sample sizes from this race were small, with seven females and 19 males taking part in the study, so relationships between performance and morphology were not explored in this group on its own. However, a brief perusal of trends among these mountain ultramarathon racers reveals that, for a given height, finishers were on average 8–8.5% heavier with 2.5% shorter legs relative to non-finishers of the same sex (unpublished data). If larger sample sizes demonstrated this to be a significant difference, it would be quite in opposition to the morphology that was associated with completion in the hot and flat conditions of the Al Andalus Ultimate Trail race.

Interestingly, the relationship between performance and thermally optimal morphology in a given set of environmental conditions may also vary by sex. In hot, dry and flat conditions, female ultramarathon runners demonstrated more widespread and pronounced relationships between performance and body size and limb proportions than males (Longman et al., 2021). Differential reproductive endocrinology and energetics between males and females is well known (Bogin, 2021; Wells, 2006), leading to characteristic patterns of sexual dimorphism in body size (Cutler, 1997; Eveleth, 1975) and body composition (Norgan, 1997; Wells, 2007) that emerge around puberty (Bogin, 2021). Across cultures, females tend to be smaller than males on average while carrying relatively less lean mass and relatively higher levels of peripheral subcutaneous fat (Taylor, Grant, Williams & Goulding, 2010; Eveleth, 1975). In endurance activities in the heat then, the smaller body size and lower lean mass for a given height of females relative to males should minimise locomotor and thermoregulatory costs, while their large reproductive fat reserves should maximise the relevant energy supply (Wall-Scheffler, 2012b; Schütz et al., 2013). Among the athletes racing in the hot-weather ultramarathon who participated in our study, 65% of the 20 females who started the race completed it, while only half of the 38 males who started the race successfully completed it (Longman et al., 2019, 2021).

Ultraendurance racing is still quite a male-dominated sport (Zingg, Knechtle, Rosemann, & Rüst, 2015), with participation rates for females typically around ~10–20% (da Fonseca-Engelhardt et al., 2013; Peter, Rust, Knechtle, Rosemann, & Lepers, 2014; Shoak et al., 2013). However, despite this much lower participation, the performance gap between male and female finishing times in ultraevents can be extremely low, from around 4–6% in ultrarunning and ultraswimming (Waldvogel, Nikolaidis, Gangi & Rosemann, 2019; Peter et al., 2014; Zingg et al., 2014) to virtually non-existent in ultra-long-distance cycling races of 400+ miles (Baumgartner, Victor Sousa, Nikolaidis, & Knechtle, 2020). Females are particularly more likely to match or even outperform men among the oldest age groups (Baumgartner et al., 2020; Stöhr et al., 2021; Waldvogel et al., 2019), the longest races (Waldvogel et al., 2019) and the races with the highest overall female participation (Knechtle, Valeri, Nikolaidis, Zingg, & Rosemann, 2016; Senefeld, Smith, & Hunter, 2016). The reasons why are not well understood, although differences in muscle characteristics, pacing strategies, oxygen and substrate utilisation, gastrointestinal physiology and endocrine function have all been implicated (for example, Tiller et al., 2021). Interestingly, when it comes to ultrarunning, nearly all semi- or self-supported events in any environment require participants to run while wearing a backpack containing all of their supplies, nutrition, water and safety equipment. This dorsal load-carrying can alter the kinematics of the trunk and lower limb and increase the cost of running (Liew, Netto, & Morris, 2017; Brown, O'Donovan, Hasselquist, Corner, & Schiffman, 2014), costs that could potentially be mitigated by the broader female pelvis, assuming similar relationships in loaded running as documented in loaded walking (Wall-Scheffler, 2012b).

### 4.4. Culture's impact on locomotor kinematics

The development of ever-more dynamic biomechanical models and experimental designs is necessary to incorporate thermoregulatory costs into our understanding of variation in structure and what it means for kinematics and locomotion in a given set of environmental and social conditions. The latter of these is perhaps more difficult to reconstruct in the past than terrain or climate, but is no less important in shaping the locomotor kinematics of a given morphology. Whether or not a behaviour is important for survival, group cohesion or play and/or is otherwise considered culturally and socially important can have an enormous impact on functional ability that is not necessarily reflected in the skeleton alone. Conversely, just because humans as a species have musculoskeletal adaptations that confer a superior capacity for sustained running (Bramble & Lieberman, 2004; Eng, Arnold, Biewener, & Lieberman, 2015; Holowka & Lieberman, 2018; Rolian, Lieberman, Hamill, Scott, & Werbel, 2009; Venkadesan et al., 2020), that does not mean that endurance running is an important part of the locomotion of all humans or that, when we do run, we are any good at it.

Both a rearfoot strike pattern, where the foot contacts the ground heel-first, and ‘overstriding’, where it does so too far out in front of the body, are considered aspects of poor running technique, as they have been shown to reduce running economy and increase the risk of injury (Daoud et al., 2012; Folland, Allen, Black, Handsaker, & Forrester, 2017; Lieberman, Warrener, Wang, & Castillo, 2015). Among non-industrialised societies for whom running is a famously important part of cultural and spiritual identity, such as the Kalenjin in Kenya and the Tarahumara in Mexico, a rearfoot strike pattern or overstriding are rare (Lieberman, 2014; Lieberman, Castillo, et al., 2015; Lieberman et al., 2010, 2020). However, this good running technique is not necessarily reflecting an evolved and naturally gifted running ability common to the human species, but rather the result of substantial learning, effort and practice. The standard approach utilised by inexperienced runners, in both industrialised and non-industrialised societies alike, seems to be to run with their natural walking kinematics: hitting the ground heel first, with the foot out in front of the body. This is documented both among non-industrialised societies in which running is uncommon, like the Daasanach of Ethiopia and Kenya and the Hadza of Tanzania (Hatala, Dingwall, Wunderlich, & Richmond, 2013; Pontzer et al., 2014), and among recreational runners in industrialised societies relative to elite competitive runners (Hasegawa, Yamauchi, & Kraemer, 2007; Larson et al., 2011). In this Special Collection, Ian Wallace and colleagues (2022) explored running prevalence and kinematics for the first time among another non-industrialised society for whom running is uncommon: the Tsimane. Their walking kinematics are explored by this team elsewhere in this Special Collection (Holowka et al., 2022). Despite their excellent lifelong cardiovascular and physical fitness, and high levels of physical activity at all ages (Gurven, Jaeggi, Kaplan, & Cummings, 2013; Kaplan et al., 2017), running is extremely uncommon: under 2.5% of their active movement over the course of three consecutive days was done at running pace. As inexperienced runners, not surprisingly they exhibited poor kinematics in unshod running trials: a rearfoot strike pattern was observed in 99% of running trials and overstriding in 98% of trials. This technique is optimal when walking, but not running; optimal running technique clearly requires learning and practice. If running is not a necessary or valuable activity in your society, this learning and practice is unlikely to happen. On the limited occasions where running is required, neither minimising risk of repetitive use injury nor maximising performance are likely to be major concerns, and suboptimal kinematics get the job done. However, if endurance running is a crucial part of your foraging strategy, is essential for survival in the environment in which you live and/or is culturally important in some way, enhancing performance and minimising injury become important. Wallace and colleagues (2022) suggest that individuals probably develop better running kinematics through both a process of unconscious natural self-optimisation and through social learning and observation.

The fact that we all share these adaptations to running, and that so too did other members of the genus *Homo*, suggests that running had a shared importance in our survival strategy at some point in our evolutionary history. Yet to run frequently and efficiently for sustained periods of time without getting injured, skeletal morphology on its own is not enough; humans must adjust their walking kinematics through social learning and practice, aided by a cultural identity that values and promotes that learning and practice. While it is impossible to know if endurance running was part of the cultural identity of past hominin and early human groups, it is quite possible that, just as its importance varies among cultures today, this may also have been the case in the past.

## 5. Concluding remarks

Making inferences about behaviour in the past from skeletal remains is challenging, given the *variability* possible in relationships between structure and function among humans, apes and probably hominins too. The nine papers in this Special Collection showcase the strength of an interdisciplinary methodology in addressing this challenge in evolutionary biomechanics, and provide a range of theoretical, comparative, experimental and modelling approaches to consider, characterise and incorporate variability in the relationship between bone form and function. Together, this work is helping to create a more nuanced framework through which to interpret morphology in the past, one that understands and incorporates diversity and complex relationships that exist between bone/body form, soft tissue, environment, culture and locomotor function.

## Research transparency and reproducibility

No data are associated with this article.
